# Matrix metalloproteinases in intestinal fibrosis

**DOI:** 10.1093/ecco-jcc/jjad178

**Published:** 2023-10-25

**Authors:** Carin Biel, Klaas Nico Faber, Ruud A Bank, Peter Olinga

**Affiliations:** Department of Pharmaceutical Technology and Biopharmacy, University of Groningen, the Netherlands; Department of Gastroenterology and Hepatology, University Medical Center Groningen, Groningen, The Netherlands; Department of Pathology and Medical Biology, University of Groningen, University Medical Center Groningen, 9713 GZ Groningen, The Netherlands; Department of Pharmaceutical Technology and Biopharmacy, University of Groningen, the Netherlands

## Abstract

Intestinal fibrosis is a common complication in patients with inflammatory bowel disease [IBD], in particular Crohn’s disease [CD]. Unfortunately, at present intestinal fibrosis is not yet preventable, and cannot be treated by interventions other than surgical removal. Intestinal fibrosis is characterized by excessive accumulation of extracellular matrix [ECM], which is caused by activated fibroblasts and smooth muscle cells. Accumulation of ECM results from an imbalanced production and degradation of ECM. ECM degradation is mainly performed by matrix metalloproteinases [MMPs], enzymes that are counteracted by tissue inhibitors of MMPs [TIMPs]. In IBD patients, MMP activity [together with other protease activities] is increased. At the same time, CD patients have a generally lower MMP activity compared to ulcerative colitis patients, who usually do not develop intestinal strictures or fibrosis. The exact regulation and role[s] of these MMPs in fibrosis are far from understood. Here, we review the current literature about ECM remodelling by MMPs in intestinal fibrosis and their potential role as biomarkers for disease progression or druggable targets.

## Graphical Abstract

**Graphical Abstract:**
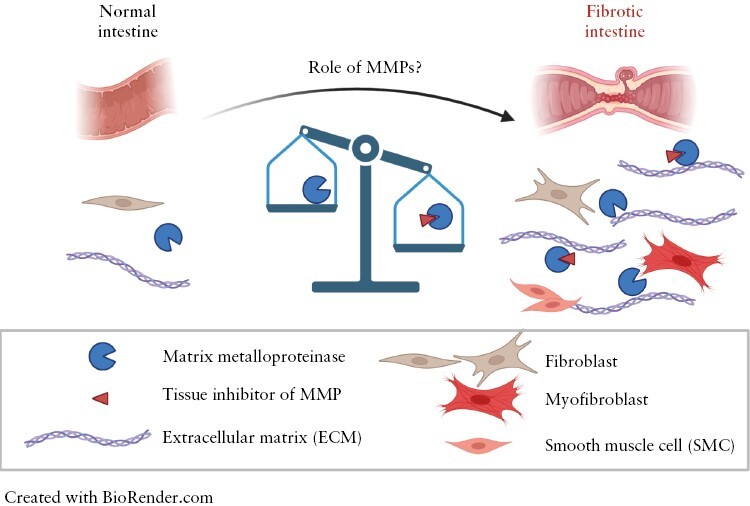

## 1. Introduction

Crohn’s disease [CD] and ulcerative colitis [UC] are inflammatory bowel diseases [IBD] characterized by relapsing and remitting inflammation of the gastrointestinal tract. The prevalence of IBD is around 0.5% in the Western world, but is increasing worldwide.^1,2^ Intestinal fibrosis/stenosis is a common complication of CD, much more than in UC.^3^ Around 30–50% of CD patients develop a stricturing disease, often around the terminal ileum in the ileocecal region, as a result of tissue fibrosis.^4–6^ To classify the patient's characteristics, the Montreal classification system has been developed. Using this system, age of onset, disease location [using L1 for terminal ileum, L2 for colon, and L3 for ileocolon] and behaviour can be classified [using B1 for nonstricturing, nonpenetrating, B2 for structuring, and B3 for penetrating disease].^7^ Current therapies target the inflammatory component of the disease by using anti-inflammatory therapy such as tumour necrosis factor [TNF]α-antagonists and corticosteroids, but these do not prevent or regress intestinal fibrosis.^8^ So far, surgery with/without stricturoplasty or endoscopic balloon dilatation are the only treatments available for intestinal fibrosis. However, these interventions cause a high patient burden and do not prevent recurrent fibrosis.^5^

The general description of tissue fibrosis, including intestinal fibrosis, is that fibrosis results from the excessive production of extracellular cellular matrix [ECM] components, produced by activated fibroblasts and other ECM-producing cell types. Besides excessive ECM production, the ECM composition and ECM remodelling are altered during fibrogenesis and fibrosis.^9,10^ ECM is the scaffolding network for cells and a substantial component of tissues. In addition to giving the tissue support, it is becoming increasingly clear that ECM also has a role in cell signalling via mechanosignalling and serves as a buffer for a plethora of signalling molecules (such as transforming growth factor [TGF]-β) and matrix metalloproteinases [MMPs].^11,12^ The ECM has a critical impact on the immune system and vice versa. For example, due to altered ECM composition during inflammation, the binding affinity of leukocytes to the ECM is increased, leading to increased retention of immune cells during inflammation.^13^ On the other hand, immune cells can produce MMPs and other ECM-degrading enzymes, especially during inflammation, which will be discussed further in this review.

Fibrosis is a universal response in different chronically diseased tissues and organs, a response aimed at tissue healing. Although the outcome, e.g. changes in ECM composition, tissue stiffening, and loss of functionality, are similar in the different organs, there are distinct mechanisms that lead to the progression of fibrosis. Furthermore, the ECM composition and remodelling are also different in different organs. While the diverse role of ECM remodelling proteins, such as MMPs and their inhibitors [tissue inhibitors of MMPs, TIMPs], was discussed previously for several organs,^14^ a dedicated review on the function of these proteins in intestinal fibrosis is still lacking. The role of ECM, MMPs, and TIMPs in intestinal inflammation in IBD has been reviewed previously^11,15,16^ but did not specifically focus on the role of the ECM remodelling proteins in the context of intestinal fibrosis. Hence, this review discusses the importance of the ECM and its remodelling proteins during intestinal fibrosis in CD, mainly focusing on the role of MMPs and TIMPs.

## 2. ECM remodelling by MMPs

MMPs play an important role in ECM remodelling during tissue homeostasis and disease.^17^ MMPs are endopeptidases that can degrade ECM components and are therefore important for ECM remodelling homeostasis.^17^ MMPs contribute to processes such as angiogenesis, cell migration, tissue repair, and inflammation. In healthy tissue, MMPs are important for normal ECM turnover, and MMP activity is very low.^15,18,19^ In IBD, MMP expression, activity, and inhibition by their inhibitors [TIMPs] are dysregulated, which is assumed to lead to serious complications such as fistula and fibrosis.^11,20^

MMPs are zinc-dependent endopeptidases that can cleave ECM constituents and other non-matrix proteins, such as cytokines, chemokines, adhesion molecules, growth factors, and their receptors. The MMP protein family shares common structural and functional elements.^17^ MMPs have a substrate specificity and are subdivided into different classes, namely collagenases [MMP-1, -8, and -13], gelatinases [MMP-2 and -9], stromelysins [MMP-3, -10, -11, and -19], matrilysins [MMP-7 and -26], membrane-type MMPs [MT-MMPs], macrophage elastase [MMP-12], and others [Table 1]. Within these classes, MMPs have their own substrate specificity and affinity. For example, MMP-1 [collagenase-1] preferentially cleaves type III collagen, MMP-8 [collagenase-2] prefers type I collagen, and MMP-13 [collagenase-3] cleaves type II collagen more efficiently.^21^ In healthy tissue, the expression of collagenases is very low, but these collagenases are upregulated during inflammation and have been linked to tissue destruction.^21^ Because MMPs are potentially hazardous for tissues, their expression, activity, and inhibition by [among others] TIMPs are tightly regulated. In most cells, MMPs are synthesized and secreted immediately. However, MMPs can be stored in granules in inflammatory cells and released upon stimulation.^15^ Every MMP, even those with similar substrate specificity, has its own expression pattern, dependent on cell type, tissue and disease context. Transcription is regulated by growth factors and cytokines and can cause 20- to 50-fold changes in MMP mRNA and protein expression upon stimulation.^22^ MMPs are synthesized as pro-peptides and need to be activated. Cleavage of the pro-peptide can be induced chemically [i.e. hypoxia or via NO], following autolytic cleavage, or via other proteases [e.g. MMPs]. Once activated by pro-peptide cleavage, MMP activity is dependent on the presence of [one of] its inhibitors [TIMPs]. In turn, TIMPs [1–4] also have their substrate specificity, but this still needs to be further elucidated.^17,19^ Finally, ECM and especially ECM-breakdown products [neo-epitopes] can also regulate MMP activity.^12^ For example, elastin peptide κ-elastin increased MMP-1 expression in human skin fibroblasts.^25^ A more detailed description of MMP regulation and substrate specificity has been described previously.^18,22^

**Table 1.** MMP classification and substrate specificity^15,21–24^

Class name	MMPs	ECM substrates	Non-ECM substrates [among others]	
			Substrate	Result
Collagenases	MMP-1, -8, and -13	Collagen types I, II, III, and V triple-helices	α1-antitrypsin Plasminogen activator inhibitor-2 α2-antiplasmin Latent TGF-β1	Inactive SERPINA1 Inactive PAI2 [SERPINB2] Inactive SERPINF2 Activated TGF-β1
Gelatinases	MMP-2 and -9	Gelatin, denatured collagens, collagen type IV	Latent TGF-β1 α1-antitrypsin Plasminogen	Activated TGF-β1 Inactive SERPINA1
Stromelysins	MMP-3, -10, and -11	ECM substrates [except triple-helical collagens]	α1-antitrypsin Plasminogen activator inhibitor-2 Endostatin Latent TGF-β1 Collagenases	Inactive SERPINA1 Inactive PAI2 Activation Activated TGF-β1 Activation
Matrilysins	MMP-7 and -26	ECM substrates [except triple-helical collagens]	α1-antitrypsin	Inactive SERPINA1
Macrophage elastase	MMP-12	Elastin	α1-antitrypsin	
Others	MMP-19, -20 -21, -23A/B, -27, and -28			
Membrane-type MMPs	MMP-14, -15, -16, -17, -24, and -25			

Besides regulation of MMP activity via transcription, translation, and proteolytic activation, MMP and TIMP expression and activity can also depend on specific DNA variants in the gene or promotor region. For example, the *TIMP-1* genotype *TIMP-1* + 372 T is significantly more abundant in CD patients compared to healthy controls. TIMP-1 protein expression in *TIMP-1* + 372 T patients is lower compared to *TIMP-1* + 372 C patients.^26^ Therefore, this *TIMP-1 + *372 T genotype might be a risk factor for CD and associated with higher MMP activity. No differences in *MMP-1*, *-2*, *-3*, *-9* and *TIMP-2* genotype distribution in IBD patients and controls were found.^26^ Although there is no significant difference between *MMP-3* genotype distribution between IBD patients and controls, distinct genotype distributions within IBD patients with different disease behaviour [e.g. stricturing or penetrating] do exist. The *MMP-3* -1613 5T5T genotype was significantly more frequent in CD patients who developed strictures, compared to the other *MMP-3* -1613 5T6T/6T6T genotypes.^26^ The MMP-3 protein also seems to be more highly expressed in IBD tissue carrying the 5T5T variant of the *MMP3* gene compared to 5T6T/6T6T genotypes, but this was not significant.^26,27^

## 3. Normal intestinal MMP expression

In the intestine, MMPs and TIMPs are expressed by different cell types in all layers of the intestinal wall [Table 2]. In normal tissue, MMPs are mostly secreted in their latent [inactive] form.^28,29^ In the lamina propria, just below the epithelial layer, expression of MMP-1, -2, -3, and -9 has been shown, as well as the expression of TIMP-1.^28,30,31^ MMP-2 expression was also detected in the muscularis mucosa,^28^ although another study did not observe MMP-2 [gelatinase A] expression in histologically normal sections.^31^ MMP-1 and -9 are shown to be expressed in the submucosa as well as in the muscularis externa.^30,31^

**Table 2.** MMP and TIMP expression in normal human intestine

Intestinal layer	Cell types	Protein
Mucosa	Epithelium	MMP-1, -2, -3, -7, -8, -9, -10, 12, -19, and -28, TIMP-2 and -3
	Villus-associated fibroblasts	MMP-11
	Lamina propria	MMP-1, -2, -3, and -9 and TIMP-1
	Myeloid cells	MMP-12
	Muscularis mucosae	MMP-2
Submucosa	Not determined	MMP-1 and -9
	Monocytes	MMP-2, -3, -9, -10, and -19 and TIMP-1
	Polymorphonuclear cells	MMP-9
	Fibroblasts	MMP-2
Muscularis externa		MMP-1 and -9
Not detected		MMP-1, -10, and -13
Not studied		MMP-14, -15 -16, -17, -23A/B, -24, -25, -26, and -27 and TIMP-3 and -4

In the intestine, epithelial and mononuclear cells [including macrophages and lymphocytes] show a more pronounced expression of MMPs and TIMPs compared to polymorphonuclear cells [including basophils, eosinophils, and neutrophils] and fibroblasts. In primary human colonic epithelial cells, high mRNA expression of *MMP-1*, *-3*, *-7*, *-10* and *-12*, and low and inconsistent expression of *MMP-2, -8, -9* were detected. *MMP-11*, *-13* and *-14* mRNA was not detected *in vitro*.^32^ Immunohistochemical staining of the normal human colon indicates that MMP-10 and -19, TIMP-2 and -3 are expressed in the epithelium.^28,33^ Immunohistochemistry revealed low levels of MMP-2 in the colonic epithelium, whereas MMP-1, -3, -7, -9, and TIMP-1 were not detected.^28,34^ Monocytes express MMP-2, while a low number of monocytes also express stromelysins [MMP-3 and -10], MMP-9, and TIMP-1. Collagenase [MMP-1, -8, and -13] expression is hardly detected in monocytes.^28,31^ In intestinal polymorphonuclear cells, only MMP-9 expression is detected.^31^ In intestinal fibroblasts, only MMP-2 expression was detected.^28^

Kirkegaard et al. did not observe any expression of MMP-1 and MMP-7 in control colon biopsies using immunohistochemistry [IHC].^28^ Using *in situ* hybridization, MMP-10 [stromelysin-2], MMP-12 [macrophage elastase], and MMP-13 [collagenase-3] were also not detected.^35^ To the best of our knowledge, expression of other MMPs and TIMPs have not been studied in the intestine.^33^ Of note, [secreted] MMPs may be hard to detect using IHC when they are not actively produced at the time of tissue fixation. Thus, in normal tissue, MMP detection using IHC might not be the most suitable detection method because MMP expression is generally very low. In this case, Western blot analysis, immunoprecipitation of tissue lysates, or mRNA analysis might be more trustworthy.^36^ RNA sequencing [RNA-seq] makes it possible to study the complete transcriptome of certain tissues in homeostasis and disease. More recently, single-cell RNA-sequencing [scRNA-seq] has been an upcoming technique that makes it possible to study cellular diversity and gene expression in a single cell type.^37,38^ scRNA-seq of normal human duodenum revealed that mature enterocytes show enriched expression of *TIMP2*, while enterochromaffin cells [an enteroendocrine cell subtype] show enriched expression of *TIMP1*.^39^ MMP transcripts are found in multiple cell types,^39^, but the number of transcripts per cell is rather low [no significant enrichment in any cell type], which is expected in a healthy gut.^40^ Smillie et al. performed scRNA-seq on colon biopsies from UC patients and healthy controls. These authors showed that MMPs and TIMPs in combination with other markers can be used as a marker for several stromal and immune cell subtypes [e.g. inflammatory fibroblasts and inflammatory monocytes]. For example, *MMP-2* appears to be a specific marker for stromal cells, and more specifically for fibroblasts. *MMP-11* was found to be a specific marker for villus-located fibroblasts [*WNT5B + 1, WNT5B + 2*], and *MMP-12* was found to be specific for myeloid cells [including macrophages].^41^

## 4. ECM composition

**Figure 1:**
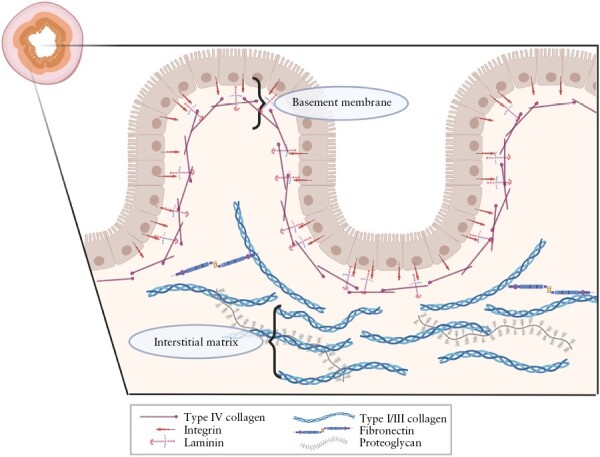
Extracellular matrix compartments of the intestine. Schematic representation of the basement membrane and the interstitial matrix. Created with BioRender.com.

The ECM is a substantial component of tissues and is essential for tissue function, architecture, and homeostasis. In the normal intestine, the ECM can be divided into two compartments, the basement membrane and the interstitial ECM [Figure 1]. The basement membrane in the intestine supports the epithelial cells and consists mainly of laminins and type IV collagen. The interstitial matrix provides tissue strength and elasticity and consists mainly of type I, III, and V collagens and elastin.^42^ ECM, besides giving mechanical support to the tissue, also regulates cell proliferation, differentiation, and migration, and has a function in cell–cell communication via integrin signalling.^43^ Moreover, the ECM also stores and releases various molecules [e.g. TGF-β] and ECM-turnover products [neo-epitopes] and thereby has a signalling function as well.^12^

**Figure 2:**
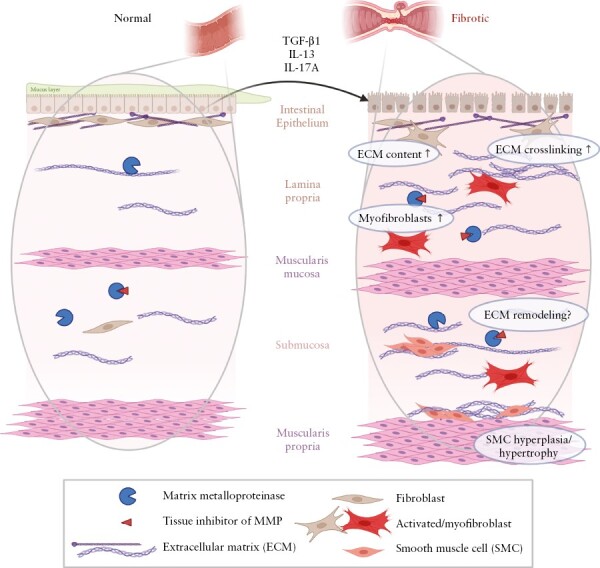
Changes in fibrotic intestine. Schematic representation of normal and fibrotic intestine. Pro-fibrotic stimuli, e.g. transforming growth factor-β1 (TGF-β1), Interleukin-13 and 17A (IL-13/IL-17A) contribute to the progression of fibrosis. Fibrosis in the intestine is characterized by increased extracellular matrix (ECM), changed ECM composition, myofibroblast differentiation, and smooth muscle cell hyperplasia and hypertrophy in the muscularis mucosae as well as in the muscularis propria. How changed in ECM remodeling contribute to intestinal fibrosis is currently unknown. Created with BioRender.com.

Fibroblasts, myofibroblasts, and smooth muscle cells [SMCs] are the main cell types responsible for ECM production and remodelling, especially during wound healing and fibrosis [Figure 2]. Fibroblasts (vimentin [VIM]^+^, α-smooth muscle actin [SMA]^−−^) are responsible for maintaining the ECM by secreting ECM components. Recent scRNA-seq studies have revealed that there are multiple fibroblast subsets and that they differ in healthy and inflamed human gut.^37,41^ For example, the inflammation-associated fibroblast subset is abundant in UC patients and can be expanded more than 100-fold compared to in healthy individuals.^41^ As a result of pro-fibrotic signalling, fibroblasts can differentiate into myofibroblasts [VIM^+^, SMA^+^]. Due to the presence of the actin cytoskeleton in myofibroblasts, these cells can contract and exert tension on the ECM. Recently, it has been shown that not only fibroblasts proliferate and migrate to the fibrotic tissue, but also SMCs [SMA^+^, DES^+^] from the muscularis mucosae and muscularis propria can produce ECM and are present in the fibrotic submucosa, while they are not present in the healthy submucosa. SMC hyperplasia and hypertrophy in the muscularis mucosae and muscularis propria [interna] in intestinal strictures seem to be the main factor contributing to the increased wall thickness.^44,45^ During wound healing, fibroblasts are attracted to the damaged tissue, start producing ECM, and use their contractile ability to initiate wound closure. During normal wound healing, ECM production and contraction will be terminated by inducing fibroblast and myofibroblast apoptosis. However, during chronic inflammation, apoptosis is inhibited/prevented and ECM production and contractility are further increased.^46,47^

In the healthy intestine, the mucosa [including the epithelium], submucosa, and muscularis propria are clearly distinguishable. In the mucosa and submucosa, collagens of the interstitial matrix [type I and III collagens] are loosely arranged and mostly present near the muscularis propria^48,49^ [Figure 1]. During chronic inflammation in IBD, the intestinal wall is damaged and requires repair and ECM reconstruction. The inflamed intestine of CD patients shows an increased type III/I collagen ratio [inflamed 0.61 vs normal 0.24 in the lamina propria] in all intestinal layers.^50^ In the inflamed fibrotic intestine, the type III/I collagen ratio increases even further [1.11 in the lamina propria].^50^ It has been shown in human tissue samples and *in vitro* in fibroblast cultures that the majority of the ECM proteins are upregulated in the fibrotic intestine resulting in an altered composition and function of the fibrotic ECM.^48–53^ ECM composition and architecture are highly influenced by post-translational modifications, which have also been shown to be affected by intestinal fibrosis^51,53^ [Figure 2]. We refer to recent reviews^42,43,54^ for more detailed overviews of the function of different collagens and ECM proteins in the intestine.

As a result of increased collagen deposition and muscle hyperplasia, the submucosa, muscularis mucosae and serosa are often expanded in fibrotic areas [Figure 2].^48,55^ In the submucosa, clusters of SMCs with associated collagen appear. Also, the luminal side of the muscularis propria contains collagenous material.^48^ Fibrosis and SMC hyperplasia/hypertrophy are different in ileal vs colonic strictures. In the ileum, muscular hyperplasia and hypertrophy are more abundant compared to the colon, while fibrosis of the submucosa and muscularis propria is more apparent in the colon.^45,47,48^ Following the increase in collagen content in the fibrotic intestine, the stiffness of the intestine is also increased [Young’s modulus of 16.7 kPa vs 2.9 kPa for healthy bowel].^56^ Tissue stiffness has a strong influence on [myo-]fibroblast behaviour. For example, intestinal [myo-]fibroblasts cultured on stiff collagen gels produce more collagen and fewer ECM-degrading enzymes,^53,56^ which further stimulates fibrogenesis.

As described above, intestinal fibrosis is characterized by thickening of all intestinal layers and increased collagen deposition. This is probably initiated by inflammation and insufficient wound healing by fibroblasts.^44^ Furthermore, ECM composition and architecture are altered as a result of dysregulated expression of collagen and collagen-crosslinking enzymes. To counteract ECM production and cross-linking, ECM degradation is mediated by proteases such as MMPs. The ECM turnover by MMPs is disturbed during fibrotic disease, thereby further promoting fibrosis.^8,56^

## 5. Increased expression of MMPs in IBD

During a clinical relapse in IBD, the mucosal architecture, including the ECM, is disrupted as a consequence of infiltrating immune cells and chronic inflammation. MMP expression and activity are generally increased in the inflamed mucosa of IBD patients [Table 3].^16,20^ We will briefly describe the role of MMPs in [intestinal] inflammation since this topic has been extensively reviewed already.^15,16,21,23,65^

**Table 3.** MMP expression in the inflamed human intestine [compared to control]

	Gene	Protein	Activity
Collagenases			
MMP-1	↑ mucosa^57^, epithelial cells^32^, fibroblasts^41^	↑ mucosa^57^, ↔^31^	↑^58^
MMP-8	↔ epithelial cells^32^	↔^31^, ↑^59^	
MMP-13		↑^60^	
Gelatinases			
MMP-2	↑ mucosa^34,57,61,62^, ↔ epithelial cells^32^	↑ mucosa^58,34,57^	↑ mucosa^28,58,29,34^
MMP-9	↑ mucosa^34,61,62^, ↑ epithelial cells^32^	↑ mostly mucosa and submucosa^28,58,31^, mucosa^29,34^	↑ mucosa^58,29^
Stromelysins			
MMP-3	↑ mucosa^38,57,61,62^, epithelial cells^32^, fibroblasts^41^	↑ mucosa^63,64,28,57^	↑^58^
MMP-10	↑ mucosa^61,62^, ↑ epithelial cells^32^		
Matrilysins			
MMP-7	↑ mucosa^61,62^, ↑ epithelial cells^32,41^		
Macrophage elastase			
MMP-12	↔ epithelial cells^32^, ↑ mucosa^35,38,62^	↑ mucosa^64^	
Membrane-bound MMPs			
MMP-14	↑ mucosa^57^		
Others			
MMP-19	↔ whole intersection^33^, ↑ mucosa^61,62^	↔, ↓, ↑ epithelial cells [all MMP-19 forms, pro-MMP-19 peptide, hinge-region MMP-19 respectively]^33^	
MMP-28	↓ mucosa^62^		
TIMPs			
TIMP-1	↑ mucosa^57,62^, ↑ absorptive and secretory epithelial cells^41^	↔ mucosa^28,64^, ↑ mucosa^58,63^	
TIMP-2	↔ mucosa^57^, ↑ immature enterocytes and mast cells^41^, ↑ mucosa^62^	↔^58^	
TIMP-3	↑^35^		

↑ = increased expression or activity in tissue from IBD patients compared to controls.

↓ = decreased expression or activity in tissue from IBD patients compared to controls.

↔ = similar expression or activity in tissue from IBD patients compared to controls.

This might be caused by changes in cellular composition [e.g. >100-fold increase in inflammation-associated fibroblasts] of the affected intestine, as well as changes in gene expression in particular cell types.^41^ Gene expression of *MMP-1*, *-2*, *-3*, *-14*, and *TIMP-1* is increased in inflamed IBD mucosa, while *MMP-9* and *TIMP-2* expression levels are not affected.^26,34,57^*In vitro* cultured colonic epithelial cells isolated from IBD patients show increased gene expression of *MMP-1*, *-3*, *-7*, *-9*, and *-10* compared to control epithelial cells.^32^ Protein expression of MMP-1, -2, -3, -9, -12, and TIMP-1, but not TIMP-2, is increased in inflamed mucosa from IBD patients compared to controls.^34,57,58,63,66^ In inflamed tissue, the presence of the latent form of MMP-1, -2, -3, and -9 is increased compared to controls.^29,58^ MMP-1, -2, -3, and -9 activity is higher in IBD mucosa compared to controls.^58^ The gene expression of *MMP-1*, *-2*, *-3*, *-14*, and *TIMP-1* is positively correlated with the histological degree of inflammation.^57^ Interestingly, only detailed characterization of the expression patterns of the different MMP-19 forms, e.g. pro-peptide and processed/activated forms, has been studied in IBD patients.^33^ Here, an antibody against the MMP-19 pro-peptide [likely to interact with inactive non-processed MMP-19] and an antibody against the MMP-19 hinge-region [detects processed and probably active MMP-19] were used. This study showed that MMP-19 pro-peptide was present in the epithelium of healthy intestinal tissue, but was not detected in IBD epithelium. On the other hand, the MMP-19 hinge-region was detected in both healthy as well as IBD epithelium. These authors concluded that MMP-19 expression is not down- or up-regulated in IBD epithelium compared to controls, but MMP-19 activation by cleavage of the pro-peptide is increased.^33^

To obtain a broader overview of changed MMP and TIMP expression, we reviewed several [sc-]RNA-seq studies that compared the transcriptome of normal and inflamed mucosa from IBD patients. Using bulk RNA-seq on inflamed and non-inflamed mucosal biopsies of UC and CD patients, Hu et al. found increased expression of *MMP-1*, *-2*, *-3, -7*, *-9*, *-10*, and *-19* and increased expression of *TIMP-1*, *-2*, and *-3* in inflamed vs non-inflamed mucosa.^61^ Furthermore, these authors showed pathway enrichment of ‘activation of matrix metalloproteinases’, ‘degradation of the extracellular matrix’, and ‘extracellular matrix organization’ in inflamed mucosal biopsies.^61^ Analysis of differentially expressed genes [DEGs] showed that *MMP-*3 is in the ‘top 10 upregulated genes’ in inflamed CD mucosa compared to healthy control mucosa [ileum and colon samples combined]. Moreover, Hong et al. showed that *SERPINE1* and *MMP-12* are significantly increased in inflamed CD mucosa compared to non-inflamed CD and healthy control mucosa,^38^ also indicating the important role of MMPs in intestinal inflammation. When DEG analysis was performed on colonic samples only, MMPs did not show up as DEGs in inflamed colonic CD mucosa compared to non-inflamed colonic CD mucosa and healthy control mucosa.^38^ This might indicate that the role of MMPs in CD inflammation is different in the ileum and colon. scRNA-seq of inflamed and non-inflamed CD mucosa revealed that *MMP-2* and *CTSK* are expressed in stromal cells, especially in activated fibroblasts.^40^ Smillie et al. showed significantly increased expression of *MMP-7* in epithelial cells of inflamed UC mucosa compared to healthy control mucosa. Moreover, these authors showed increased expression of *TIMP-1* by absorptive epithelial cells and secretory epithelial cells and increased expression of *TIMP-2* by immature enterocytes in inflamed UC mucosa compared to healthy controls. Furthermore, fibroblasts had higher expression of *MMP-1* and *-3* and mast cells had higher expression of *TIMP-1* in UC mucosa compared to healthy controls.^41^ Interestingly, Smillie et al. found that the inflamed and uninflamed UC mucosa DEG signature was very similar, indicating that uninflamed UC mucosa is still affected by UC inflammation.

Increased activity of MMPs is also reflected by MMP-degradation products in the serum of IBD patients. C1M [type I collagen degradation product of MMP-2, -9, and-13], C3M [type III collagen degradation product of MMP-9], C4M [type IV collagen degradation product of MMP-2, -9, and -12], and C6Ma3 [type VI collagen alpha chain degradation product of MMP-2 and -9] are significantly elevated in the serum of Montreal B1 [non-stricturing, non-penetrating] CD patients compared to healthy controls.^67,68^ In contrast, C5M [type V collagen degradation product of MMP-2 and -9] and C6M [type VI collagen degradation product of MMP-2 and -9] levels are similar between Montreal B1 CD patients and healthy controls.^68^

Interestingly, when comparing MMP expression in different locations in the intestine, it seems that MMP expression and activity are lower in CD patients with ileum involvement compared to CD patients with colonic inflammation. De Bruyn et al. showed that *MMP-12* expression is lower in mucosa from inflamed CD ileum compared to the colon, whereas other MMPs and TIMPs were not differently expressed.^62^ Mortensen et al. showed that BGM [MMP-3 and -9 degradation product] was lower in the serum of CD patients with ileum [Montreal L1] vs colonic [Montreal L2/3] involvement.^69^ It might be possible that fibrostenotic complications develop more often in the ileocecal region because there is a lower ECM-degrading capacity at that location, but this should be further investigated.

Increased expression of MMPs during inflammation has several consequences. MMPs might increase cellular migration by loosening the cells from each other and/or the surrounding ECM, thereby promoting the infiltration of immune cells and fibroblasts. Furthermore, MMPs can activate cytokines and chemokines, thereby promoting chemotaxis and/or activation of inflammatory cells. Migration of inflammatory cells and fibroblasts, on the one hand, might promote wound healing as these cells are needed for a proper wound healing response. However, when dysregulated MMP activation also causes defects in the epithelial barrier and inflicts too much tissue damage, there might be a vicious circle of MMP activation and inflammation promotion.

## 6. MMP expression in CD patients is lower than in UC patients

Remarkably, while both CD and UC patients have a generally increased expression and activity of MMPs, patients with CD develop strictures much more often than UC patients.^5^ Therefore, we reviewed in more detail whether there are differences between the expression of MMPs and TIMPs in CD compared to UC patients [Table 4].

**Table 4.** Expression and activity of MMPs and TIMPs in CD tissue compared to UC tissue

	Gene	Protein	Activity
Collagenases			
MMP-1	↔^57^, ↓^70^		↓^58^
MMP-13	↑^35^	↓^60^	
Gelatinases			↓^71^
MMP-2	↓^57^	↓^34^	↔^34^, ↑^72^
MMP-9	↓^70^	↔^34^	↓^34,72^
Stromelysins			
MMP-3	↓^57,58^	↔^63^	
MMP-10	↔^35^		
Macrophage elastase			
MMP-12	↔^35^, ↓^70^		
Membrane-bound MMPs			
MMP-14	↔^57^		
TIMPs			
TIMP-1	↔^57^	↔^63^	
TIMP-2	↔^57^	↔^58^	

↑ = increased expression or activity in tissue from CD patients compared to UC tissue.

↓ = decreased expression or activity in tissue from CD patients compared to UC tissue.

↔ = similar expression or activity in tissue from CD patients compared to UC tissue.

In inflamed CD mucosa, there is a lower gene expression of *MMP-2* and *-3* compared to UC, whereas *MMP-1* and *-14* and *TIMP-1* and *-2* are not differently expressed.^57^ Lawrance et al. showed that gene expression of ECM proteins and several MMPs [*MMP-1*, *-3*, *-9*, and *-12*] were overexpressed in full-thickness colon tissue of UC patients compared to CD patients.^70^ A slightly lower MMP-2 and -9 protein expression and activity was found in CD vs UC intestinal mucosa, but this was not significant.^34^ RNA-seq of UC and CD mucosa did not reveal differences in MMP or TIMP gene expression.^61^ Similar results have been obtained by Bailey et al., in which they also showed an increased MMP-2 and a decreased MMP-9 expression in both mucosa and muscularis of uninflamed CD intestine compared to UC intestine.^55^ Total MMP-1 activity was found to be lower in CD mucosa compared to UC, whereas TIMP-2 expression was higher.^58^ Baugh et al. also showed a lower gelatinase [MMP-2 and -9] expression in CD compared to UC.^29^ Another study found that MMP and TIMP expression and activity were very similar between UC and CD mucosa, and only MMP-1 activity was lower and TIMP-2 protein expression was higher in CD mucosa compared to UC mucosa.^26^ In CD mucosal explant cultures, protein expression of *active* MMP-1, -2, and -3 was lower compared to UC mucosal explant cultures.^27^ Differences in serum levels of ECM degradation products were also found between CD and UC patients.^72^ VIMC [vimentin degradation product of MMP-2 and -8] levels are higher in CD patients compared to UC patients, while C3M and BGM levels are higher in UC patients.^72^ No differences between serum levels of C1M, C4M, and C5M were found in CD vs UC patients.^73,74^

The results described above show an altered expression and activity of ECM remodelling MMPs in CD compared to UC patients. Interestingly, CD patients, who are more prone to developing fibrosis, show a generally lower expression and activity of MMPs compared to UC patients, who are less prone to developing fibrosis. Whether lower MMP activity in CD vs UC patients indeed is [one of] the reasons for the higher prevalence of fibrosis in CD patients is not known. What should be taken into account is that MMP expression and activity are generally lower in the ileocecal region compared to the colon.^62,69^ Since ileocecal inflammation is often present in CD patients, while UC is characterized by colonic inflammation, direct comparison between these two patient groups might fall short.^5^ In CD patients, stenosing and stricturing disease is more often present in patients with ileal or ileocolonic disease compared to patients with colonic involvement only.^75,76^ Stricture development thus seems to be location-specific. Since the luminal diameter of the terminal ileum is smaller than other parts of the intestine, stricture development might be symptomatic earlier in this region compared to other intestinal regions.^6,77,78^

Differences in MMP regulation between CD and UC patients might also be explained by differences in the type of inflammation [Table 5]. Inflammation is limited to the mucosa in UC, while CD inflammation is transmural. Moreover, CD is characterized by a Th1 immune response, while UC seems more Th2-driven.^73^ Although this separation is debatable, several studies have shown a higher expression of IFN-γ [Th-1 cytokine] in CD tissue compared to UC and healthy tissue, while IL-13 [Th-2 cytokine] is expressed at similar or lower levels in CD tissue compared to UC tissue.^73^ CD is also characterized by higher IL-17A levels, indicating active Th-17 inflammation as well.^73,79^ This results in the expression and activity of different cytokines, which may alter MMP expression and activity.

**Table 5.** *In vitro* effect of Th-1, -2, and -17 cytokines on MMP expression

	In vivo		In vitro	
	Pro-/anti-fibrotic	Expression in intestinal fibrosis	mRNA	Protein
Th-1 cytokines				
IFN-γ	Anti-fibrotic		↑ MMP-1; ↔ MMP-9 and TIMP-1	
TNF-α	Pro-fibrotic		↑ MMP-1; ↔ MMP-9 and TIMP-1	↑ MMP-1
Th-2 cytokines				
IL-4	Pro-fibrotic	Not studied	↑ MMP-1; ↓ MMP-9	
IL-13	Pro-fibrotic	↑ muscle	↔ MMP-1; ↓ MMP-9	↓ MMP-1 and -2
Th-17 cytokines				
IL-17A	Pro-fibrotic	↑ muscle	↑ MMP-1; ↔ MMP-9 and TIMP-1	↑ MMP-1, -3, and -12 and TIMP-1
IL-22	Pro-fibrotic		↔ MMP-1 and -9 and TIMP-1	
IL-23	Pro-fibrotic		↑ MMP-1 and -9 and TIMP-1	
Treg				
IL-10	Anti-fibrotic	Not studied	↑ MMP-1; ↔ TIMP-1	

↑ = increased expression compared to control.

↓ = decreased expression compared to control.

↔ = similar expression compared to control.

The Th-1 cytokine IFN-γ alone does not influence MMP-9 expression *in vitro* cultured intestinal fibroblasts but suppresses TNF-α- and IL-1α-induced expression of MMP-9.^80^ In intestinal epithelial cells, IFN-γ induces expression of TIMP-.^80^ Interestingly, the Th-2 [but not Th-1] cytokines IL-4 and IL-13 and the Th-17 cytokine IL-17A promote intestinal fibrosis development.^71,74,81^*IL-13* gene expression is upregulated in the muscle of CD strictures,^55^ but no differences in IL-13 production of muscularis explants overlying strictures were found.^82^ On the other hand, both studies found higher expression of IL-13Rα1 in isolated cells from tissue overlying strictures compared to control tissue.^55,82^ Stimulation of intestinal muscle fibroblasts causes decreased pro-MMP-1 and -2 synthesis.^55^ Filidou et al. also showed that Th-2 cytokines [IL-4 and IL-13] decreased *MMP-9* mRNA levels in intestinal myofibroblasts, but also showed that IL-4 alone increased *MMP-1* gene expression.^83^

Expression of the Th-17 cytokine IL-17A is increased in fibrotic muscle vs non-fibrotic muscle of CD patients.^84^ Stimulation with IL-17A causes an increase in MMP-3 and -12 and TIMP-1 expression in fibroblasts isolated from fibrotic, non-fibrotic, and control intestine.^84,85^ Other Th-17 cytokines, e.g. IL-22 and IL-23, have different effects on intestinal myofibroblasts. While IL-22 did not affect MMP-1 or TIMP-1 expression, IL-23 induced MMP-1 and TIMP-1 expression in intestinal fibroblasts.^83^

Thus, it seems that there is a different regulation of MMPs and TIMPs in CD vs UC, which might be caused by a different type and/or location of intestinal inflammation. Unfortunately, there is little literature on the role of MMPs in different regions and layers of healthy and IBD intestines and how these could promote intestinal fibrosis.

## 7. MMP expression in intestinal fibrosis

MMPs have diverse roles in organ fibrosis. In the first place, it is logical to assume that MMP activity is lower during fibrosis development and established fibrosis, as this would explain excessive ECM accumulation. However, MMPs can also have a pro-fibrotic role, for instance through activating TGF-β by MMP-2 and -9 or by promoting epithelial–mesenchymal transition [EMT] by MMP-3.^14,86^ In the next section, the current literature on MMP and TIMP expression and activity in fibrotic vs inflamed or control CD specimens is reviewed [Table 6].

**Table 6.** Expression and activity of MMPs and TIMPs in fibrotic intestine compared to non-fibrotic control tissue

	Gene	Protein	Activity
Collagenases			
MMP-1	↑^30^ ↑^51^	↑ submucosa and muscularis^30^, ↑ muscularis and mucosa^55^, ↔ mucosa^30^, inflamed fibrotic mucosa^58^, ↔^31^	↔ inflamed fibrotic mucosa^58^
MMP-8		↔^31^	
Gelatinases			
MMP-2		↔ inflamed fibrotic mucosa^58^	↑ mucosa^55^, ↔ inflamed fibrotic mucosa^58^, ↔ muscularis^55^
MMP-9	↔^30^	↑ mostly mucosa and submucosa^31^, ↔ inflamed fibrotic mucosa^58^	↔muscle and mucosa [increased compared to cancer control]^55^, ↔ inflamed fibrotic mucosa^58^
Stromelysins			
MMP-3	↑ ^28, 54^	↑ mucosa [less pronounced], submucosa and muscularis^30^ ↔ inflamed fibrotic mucosa^58^	↔ inflamed fibrotic mucosa^58^
Membrane-bound MMP			
MMP-14	↑^51^		
TIMPs			
TIMP-1	↔,^30^ ↑^51^	↑ muscle^55^, ↔ mucosa^55^, ↔inflamed fibrotic mucosa^58^	
TIMP-2		↔ inflamed fibrotic mucosa^58^	

↑ = increased expression or activity in fibrotic tissue from CD patients compared to control tissue.

↓ = decreased expression or activity in fibrotic tissue from CD patients compared to control tissue.

↔ = similar expression or activity in fibrotic tissue from CD patients compared to control tissue.

No differences in expression and proteolytic activity of MMP-1, -2, -3, and -9, nor in the expression of TIMP-1 and -2 were detected between inflamed fibrotic CD mucosa and inflamed non-fibrotic CD mucosa.^26^ In contrast, others found differences when non-inflamed fibrotic CD tissue was compared to control or inflamed CD tissue. In general, it was found that TIMP-1 expression is increased in the fibrotic [both mucosa and muscularis] intestine compared to non-fibrotic CD intestine and non-IBD controls.^55,66^ Interestingly, *MMP-1* and *-14*, as well as *TIMP-1* expression, were significantly increased in full-thickness fibrotic terminal CD ileum when compared to non-fibrotic and non-CD controls.^51^ Warnaar et al. also found that *MMP-1* and *MMP-3* are upregulated in muscularis and submucosal tissue of the fibrotic intestine. MMP-1 was not differentially expressed in the mucosa, but increased in the submucosa and muscularis of stenotic CD intestine compared to controls, while MMP-3 expression was increased in all intestinal layers.^30^ Pro-MMP-1 was shown to be increased in both muscularis and mucosa of the fibrotic CD intestine compared to controls and inflamed intestine. MMP-9 activity was not altered whereas MMP-2 activity was increased in the mucosa, but not in the muscularis.^55^ Bailey et al. detected higher expression of stromelysins [MMP-3 and -10] in areas of SMC proliferation, suggesting a pro-fibrotic role for these MMPs by promoting migration and the ability of SMCs to invade the submucosa.^31^ Altogether, it appears that the expression and activity of several MMPs [collagenases, gelatinases, as well as stromelysins] are increased in fibrotic intestinal tissue compared to non-fibrotic tissue. However, at the same time, TIMP-1 expression is also increased^51,55,66^ and thus could counteract the increased MMP expression *in vivo*. Unfortunately, the expression of MMPs is not well studied in layers of the human intestine other than the mucosa.

As a measure of the formation and degradation rates of ECM, ratios between MMPs, TIMPs, and collagens have been used. Warnaar et al. showed that *MMP-1*/*TIMP-1* and *MMP-3*/*TIMP-1* ratios were increased in fibrotic terminal ileum compared to controls, but similar to proximal resection margins. *MMP-9*/*TIMP-1* was found to be similar in fibrotic terminal ileum compared to controls,^30^ suggesting that ECM breakdown may take place at the same pace in fibrotic vs non-fibrotic regions. Possibly, the increased collagen production is not compensated for by increased ECM breakdown. Indeed, the MMP-1/collagen synthesis ratio was found to be lower in fibrotic muscularis compared to uninflamed CD and inflamed UC.^55^ This indicates that the increased MMP expression is not sufficient to compensate for the increased collagen expression.

The expression of MMP and TIMP genes and proteins does not translate 1-to-1 to their actual activity in the tissue, since this is highly regulated at a post-translational level. To determine the balance of ECM remodelling more closely, the ratio between ECM formation products and ECM degradation products by MMPs can be determined. Interestingly, when formation and degradation products of type I, III, V, and VI collagen were determined in the serum of CD patients, the balance of type I and III collagens in CD patients with stricturing disease [Montreal class B2] pointed more towards collagen degradation when compared to healthy controls. On the other hand, type V collagen formation/degradation balance was more towards formation in CD patients with stricturing disease compared to healthy controls, while the type VI balance was unchanged.^68^ Bourgonje et al. also measured serum levels of Pro-C3 and C3M, but in contrast to van Haaften et al. did not find a difference in C3M/Pro-C3 ratio in the serum of Montreal B2 CD patients compared to healthy controls. They did find an increase in Pro-C4/C4M ratio, C1M, and C6Ma3 serum levels in Montreal B2 CD patients compared to healthy controls.^67^ A reason for these different results might be the difference in patient inclusion/exclusion criteria. For example, van Haaften et al. only included CD patients with ileal disease [L1], while Bourgonje et al. did not specifically include or exclude patients with different disease locations. Interestingly, it has been shown previously that disease location might influence serum levels of another MMP-degraded ECM protein [BGM].^69^ It might be difficult to specifically find differences between Montreal B2 [stricturing], B1 [inflammatory], and healthy controls since the formation and degradation products were measured in patients’ serum, which does not necessarily represent the activity at the specific part of the intestine. It is therefore possible that inflammation in other parts of the CD intestine masks the alterations at the fibrotic region.^68^ When B1 and B2 phenotypes are compared, only the collagen IV formation rate [ProC4/C4M ratio] appears different [increased] in the serum of B2 patients compared to B1 patients.^67^ Together, these results suggest higher MMP-2, -9, -12, and/or -13 activity in CD patients with stenosis.

## 8. *In vitro* studies

Fibroblasts are considered to be the main cell type involved in ECM production and remodelling during fibrosis and fibrogenesis. MMP expression of fibroblasts has been studied from three different perspectives, namely the fibroblast source, culture conditions, and exposure to soluble factors [pro-fibrotic molecules]. Of note, it is not always clear whether the authors used fibroblasts or myo-fibroblasts since these terms are not consistently used between the various papers. However, as described before, many fibroblast subtypes exist and one subtype might [and can] differentiate into another, also during culture. Depending on culture conditions, ‘spontaneous’ differentiation from fibroblasts [α-SMA^−^] to myofibroblasts [α-SMA^+^] does occur in culture and is not always mentioned or verified in scientific publications. This is probably the case when culturing isolated fibroblasts from the normal, or healthy intestine since α-SMA is normally only detected in the muscularis mucosae and muscularis propria, and not in areas with fibroblasts [epithelial lining, lamina propria, or submucosa]^45,87^

First, MMP and TIMP expression is different between control, inflammatory, and fibrotic tissue-derived fibroblasts [Table 7].^66,84^ De Bruyn et al. showed lower expression of *MMP-3*, *-10*, *-11* and *-24*, and increased expression of *MMP-2* and *TIMP-1* and *-2* in myofibroblasts from stenotic tissue compared to non-stenotic myofibroblasts, while no difference was observed for *MMP-1*, *-16*, and *-17* expression. Moreover, MMP-2 and MMP-3 activity were higher in stenotic myofibroblasts compared to inflamed and normal myofibroblasts.^53^ McKaig et al. showed significantly increased expression of TIMP-1 by stenotic myofibroblasts from CD patients compared to UC myofibroblasts and control myofibroblasts, but they did not detect differences in *MMP-1*, *-2*, *-3*, and *-9* expression.^88^ Thus, myofibroblasts isolated from healthy, inflamed and stenotic regions of the intestine are different and express MMPs and TIMPs differently. MMPs can be either higher or lower expressed in fibrotic myofibroblasts compared to normal myofibroblasts, whereas TIMPs are more highly expressed in stenotic myofibroblasts. This indicates that there is in general lower ECM degradation activity by myofibroblasts from stenotic regions compared to myofibroblasts from the healthy or inflamed regions.

**Table 7.** *In vitro* MMP and TIMP expression

Fibroblast source	Comparison	mRNA	Protein	Activity
Stenotic	Stenotic vs non-stenotic fibroblasts	↔ MMP-1, -16, and -17^53^; ↓ MMP-3, -10, -11, and -24^53^; ↑ MMP-2 and TIMP-2^53^; ↑ TIMP-1^66,88^	↓ MMP-12^66,84^; ↔ MMP-3^66,84^; ↑ TIMP-1^66,88^; ↔ TIMP-1^84^; ↔ TIMP-2^88^	↓ MMP-12^66^ ↑ MMP-2 and -3^53^
Normal	Plastic vs matrix cultured fibroblasts	↑ MMP-3^89^		↑ MMP-2
Ccd-18Co	Plastic vs matrix cultured fibroblasts	↑ MMP-3		↑ MMP-2^71^
Normal	Increasing stiffness			↑ MMP-3^53^
Stenotic	Increasing stiffness			↓ MMP-3^53^
Ccd-18Co	Increasing stiffness	↓ MMP-1 and -3^56^		↓ MMP-3^53^

↑ = increased expression or activity.

↓ = decreased expression or activity.

↔ = similar expression or activity.

Second, the influence of culture substrate and stiffness on MMP and TIMP expression has been studied *in vitro*. One reason for the different expression patterns *in vitro* compared to *in vivo* is the substrates on which intestinal fibroblasts are cultured. Fibroblasts are usually cultured on plastic or glass with a stiffness in the GPa range,^90^ while the stiffness of a healthy bowel is around 2.8–4.3 kPa and that of a stenotic bowel 28–30 kPa.^53,91^ Fibroblasts can react to different substrates and matrix stiffness via focal adhesions [FAs]. FAs are protein complexes that attach the intracellular cytoskeleton via transmembrane proteins, and integrins, to the ECM.^92^ Focal adhesion kinase [FAK], one of the FA proteins, is more highly expressed in fibroblasts isolated from the fibrotic intestine compared to controls.^93^ FAs are also known to be upregulated in fibroblasts grown on a stiff matrix, compared to fibroblasts grown on a soft matrix, and in myofibroblasts exposed to type XVI collagen.^74,91^ Subsequent downstream FA signalling [via Rho/ROCK] can be pro-fibrotic and also influence MMP and TIMP expression. Indeed, when Ccd-18Co fibroblasts [a human colonic fibroblast cell line] are cultured in a collagen I, hyaluronic acid, or fibronectin gel or agar, instead of normal plastic, this results in higher expression and activation of MMP-2.^71^ Moreover, when Ccd-18Co fibroblasts are cultured on acrylamide gels, *MMP-1* and *MMP-3* gene expression decreases with increasing gel stiffness, with the highest MMP expression on gels with a physiologically normal stiffness [4.3 kPa] and the lowest expression in gels with stiffness corresponding to that of the stenotic bowel [28 kPa].^56^ Primary intestinal fibroblasts also show upregulation of *MMP-3* when cultured in decellularized human intestinal scaffolds compared to fibroblasts cultured on plastic.^89^ Interestingly, de Bruyn et al. showed that primary myofibroblasts isolated from normal, inflamed only, or stenotic intestines respond differently to matrix stiffness. As expected, when cultured on a stiff collagen-coated matrix, normal myofibroblasts increase their MMP-3 activity. In contrast, myofibroblasts from the stenotic intestine decreased their MMP-3 activity on a stiff matrix. These authors showed a similar response to matrix stiffness in Ccd-18Co fibroblasts, suggesting that these cells represent stenotic fibroblasts.^53^ Taken together, the expression and activation of MMPs are dependent on the [myo-]fibroblast source, but also on the environment in which they are cultured. In future studies, fibroblast sources, as well as fibroblast culture substrate, should be carefully chosen and documented. To set up good *in vitro* models using intestinal fibroblasts, proper comparisons between *in vitro* and *in vivo* expression of MMPs and TIMPs in different circumstances should be made.

Lastly, fibroblasts are cultured in the presence of pro-fibrotic factors to delineate the molecular mechanisms involved in intestinal fibrosis. Here we will focus on TGF- β1 and its role in MMP and TIMP regulation in intestinal fibroblasts.

TGF-β1 is upregulated in the fibrotic intestine and isolated fibroblasts [Table 8].^66,84^ It is well known that TGF-β1 plays an important role in the wound healing response and that TGF-β1 acts as a pro-fibrotic signalling molecule. Also in the intestine, TGF-β isoforms [1, 2, and 3] are differentially expressed in CD mucosal samples and isolated fibroblasts.^81,94^ In particular, TGF-β1 and TGF-β3 mRNA were more abundant in macrophages and fibroblasts in the lamina propria of CD intestines compared to normal intestines.^81^ In the fibrotic CD intestine, the protein expression of TGF-β1 is increased in all layers of the intestine compared to inflamed UC as well as control intestines.^50^ Interestingly, in primary fibroblasts from CD patients with stenosis, TGF-β1 and TGF-β2 expression is increased, while TGF-β3 expression is decreased compared to normal fibroblasts.^94^ When normal fibroblasts are treated with TGF-β1 or TGF-β2, TIMP-1 expression is increased, while treatment with TGF-β3 does not affect TIMP-1 expression, suggesting a pro-fibrotic role for TGF-β1 and TGF-β2.^88^ No effect of TGF-β on MMP-1, -2, -3, and -9 expression was found in Ccd-18Co intestinal fibroblasts.^71^ In conclusion, TGF- β1 might influence decreasing ECM breakdown, by increasing the expression of TIMPs.

**Table 8.** Role of TGF-β *in vitro* expression of MMPs and TIMPs

	In vivo	In vitro		
	Pro/anti-fibrotic	Expression in intestinal fibrosis^a^	mRNA^b^	Protein^b^
TGF-β1	Pro-fibrotic	↑	MMP-1 ↔; TIMP-1 ↑	MMP-2 and TIMP-1 ↑; MMP-3 ↔; MMP-9 ↑; MMP-12 ↓
TGF-β2		↑		TIMP-1 ↑
TGF-β3	Anti-fibrotic	↓		TIMP-1 ↔

↑ = increased expression compared to control.

↓ = decreased expression compared to control.

↔ = similar expression compared to control.

^a^Compared to non-fibrotic control.

^b^Compared to non-treated control.

A major limitation of *in vitro* studies using [myo-]fibroblast cultures is that freshly isolated primary [myo-]fibroblasts are different from [myo-]fibroblasts cultured *in vitro* for longer periods [higher passage] Moreover, isolation methods influence the yield and type of fibroblasts that are isolated.^95,96^ The absence or presence of an advanced culture substrate, stress/strain, flow/shear, topography, and specific stiffness does influence the cellular response to stimuli.^90,97,98^ For example, the *ACTA2* and *TGF-β1* gene expression of primary intestinal myofibroblasts cultured in two dimensions [2D] [conventional plastic] did not change upon stimulation with TGF-β1, while the same myofibroblasts cultured in decellularized duodenum matrix did show a significant increase in *ACTA2* and *TGF-β1* gene expression upon TGF-β1 stimulation, showing the importance of the presence of a 3D environment.^89^ Moreover, using primary intestinal fibroblasts, it has been shown that the stiffness of the culture substrate influences fibroblast morphology, proliferation rate, and expression of genes involved in matrix turnover, showing the importance of substrate stiffness.^91^ Fibroblast source [cell line, primary, healthy, inflamed, or stenotic] is of great influence on how fibroblasts respond to, for example, matrix stiffness.^53^ However, using mRNA expression analysis, these authors showed that, even when using the same selection criteria, isolation, and culture methods, gene expression between primary myofibroblasts from different patients shows different patterns.^53^ Currently, it is not known what would be the most representative culture method for the best translation of *in vitro* studies to the *in vivo* situation. Advanced models would probably have a better translational potential, but these models are usually more labour-intensive, more costly, and less robust than conventional models. Therefore, the model of choice depends on the research question. Moreover, researchers should be aware of the limitations that come with the experimental model they apply.^99^ Comparisons between available *in vivo* data and *in vitro* data will be necessary to validate the best models. However, as noted in the next section, we should further improve the data obtained from human studies.

## 9. Limitations of patient studies

As described above, apparent contradictory results have been described for MMP expression, localization, and activity in the healthy and fibrotic intestine. There are several reasons to explain this. First, the expression, localization, and activity of MMPs are determined in samples obtained from patients using different inclusion/exclusion criteria, from different locations in the small or large intestine, or from a different layer of the intestinal tissue. Control and affected tissues [inflamed, fibrotic, or both] are very heterogeneous, and not specified well in every study. In the case of the fibrotic intestine, the tissue might originate from the jejunum, ileum, or colon and can be inflamed or non-inflamed. This is not always described in detail, which makes a direct comparison between different studies difficult. Moreover, healthy tissue used as controls was obtained either from macroscopically or histologically non-affected resection margins of CD patients,^30,51,55,58,64^ and from non-CD controls with another underlying disease [UC or cancer].^28,30,31,51,55,56^ Microscopically normal intestines might still be affected by inflammatory conditions proximal to the normal-appearing intestine, as shown by Baugh et al., who showed that the expression and activation of MMPs were increased in both non-inflamed as well as inflamed IBD mucosa compared to controls.^29^ Moreover, histologically normal intestine from patients undergoing bowel resection due to colon/pancreatic cancer is very often used as a non-IBD control. However, tissue under the influence of cancer also has impaired ECM remodelling. Moreover, these patients may have undergone [chemo]therapy before surgery,^17,100^ which will also affect the intestinal mucosa. Second, MMP expression and activity vary between anatomical locations of the gut and between different tissue layers, again making a direct comparison between studies complicated. Third, current and earlier [drug] treatments of IBD patients might affect the expression and activity of MMPs and TIMPs. For instance, it has been shown that MMP-7, -9 and -26 and TIMP-1 and -3 are downregulated in specific cell types in patients after using immunosuppressive drugs.^101^ De Meijer et al. also showed downregulation of MMP-1 and -3 in mucosa after *ex vivo* exposure to infliximab, a commonly used anti-TNF-α-drug.^27^ Surprisingly, medication use is usually not addressed in the patient selection or taken into consideration in the data analysis. Lastly, the expression and activation of MMPs is very complex and tightly regulated.^18,22^ Contradictory results in MMP expression, activation, or localization using different analytical methods for MMP detection is therefore not surprising. It is thus important to clarify, especially when using antibodies against MMPs and TIMPs, which variant of the protein [pro-peptide or active form] is actually detected. Most of the above-described studies performed Western blot analyses, IHC, or ELISA using uncharacterized or unspecified antibodies. Using such antibodies, it is possible to detect other or fewer MMP conformations than assumed. Unfortunately, only one study has reported a direct comparison between different antibodies [against MMP-19 pro-peptide and MMP-19 hinge-region] detecting either pro-MMP-19 [before activation] or total MMP-19 [before and after activation]. When using these two antibodies, different expression patterns in IBD intestines were found, highlighting the importance of using well-defined detection methods.^33^

## 10. MMPs as drug targets

Depending on the pro- or anti-fibrotic role of a specific MMP, MMPs are considered as potential drug targets or therapeutic agents to treat intestinal fibrosis, for example by stimulating ECM breakdown or inhibiting cytokine and growth factor activation. Specific MMP antagonists are available, and other drugs do interfere with MMP and/or TIMP expression and activation. MMP inhibition can be obtained via several approaches, such as [broad-spectrum] small-molecule inhibitors, anti-MMP-antibodies, or micro-RNAs.^17,102^ In the context of intestinal fibrosis, only anti-MMP-9 antibodies have been evaluated as a potential treatment option.^103^ Indeed, treatment with anti-MMP-9 antibodies in an intestinal fibrosis mouse model resulted in less obstruction and better preservation of villi compared to isotype controls. Moreover, lower amounts of hydroxylated proline in collagens were found in anti-MMP-9-treated intestinal grafts compared to isotype controls.^103^ Unfortunately, measurements of MMP-9 activity in Montreal B2 CD patients have not yet been conclusive, since studies showed either an increase in all Montreal classes [B1, 2, and 3] in C3M serum levels compared to healthy controls^68^ or no difference between Montreal B2 and controls.^67^ Thus, MMP-9 activity might be more related to inflammation or CD in general rather than fibrosis. MMP-9 antagonists have been tested in CD and UC patients in several clinical trials, but these resulted in conflicting data due to differences in study endpoints, patient characteristics, and a small number of patients.^104–107^ It was suggested that MMP-9 antagonists might be favourable in specific patient groups. Still, more [human] pre-clinical evidence for the potential benefits of MMP-9 antagonists in intestinal fibrosis should be generated, since this has barely been studied so far.^104^ More generally, effective treatment by targeting MMPs will only be possible when the expression, activity, and function of the targeted MMP[s] is fully known. Moreover, the timing, location, and specificity of the doses might be crucial in the ultimate beneficial or harmful effect of such treatment.^17,102^

Currently used IBD medication has been shown to influence MMP expression and activity. In mucosal explant cultures, infliximab, an anti-TNF-α antibody, decreases MMP-1, -3, and -9 and TIMP-1 secretion. Interestingly, only tissues from patients with certain genotypes seemed to show decreased expression of these MMPs upon infliximab treatment.^27^ In biopsies of patients after anti-TNF-α treatment, the expression of MMP-7, -9, and -26 and TIMP-1 and -3 was lower compared to biopsies before treatment.^101^ At the gene expression level, decreased expression of almost all MMPs and TIMP-1 and -2, and increased expression of MMP-28 in CD patients after infliximab treatment has been shown.^62^ In the serum of patients treated with vedolizumab [an anti-α4β7-integrin] lower levels of C1M, C3M, C4M, and C6Ma3 were detected, indicating a lower activity of MMPs [MMP-2, -9, -12, and -13].^108^ Decreasing MMP activity in the above-mentioned cases [TNF-α- or α4β7-integrin inhibition] is probably caused by the anti-inflammatory role of these therapeutics. To the best of our knowledge, the effect of MMP activity by other commonly used CD treatments [mesalazine, azathioprine, corticosteroids] in CD patients has not yet been studied.

Interestingly, a retrospective study found that when immunomodulating drugs [azathioprine or anti-TNF-α therapy] are given early enough [≥6 months before first surgery] in CD patients, this can delay, but not prevent, disease phenotype changes [Montreal B1 to B2/3] and the time to the first surgery [which is usually needed for stricturing or penetrating disease].^109^ Unfortunately, when treatment is started [too] late, the need for intestinal surgery can no longer be reduced.^8,109^ Thus, as mentioned above, the timing of treatment to prevent intestinal fibrosis, either via direct or indirect targeting of MMPs, is very important. Early diagnosis, or prediction of changing disease phenotype using biomarkers, such as MMP-degraded collagens, could be helpful.

## 11. Concluding remarks

The specific roles of MMPs and TIMPs in intestinal fibrosis remain unclear, although some conclusions can be drawn. In general, gene expression of various MMPs is increased, or at least similar, in the fibrotic intestine in CD patients as well as *in vitro* models, while at the same time TIMP-1 is overexpressed. While overexpression has been shown for a few MMPs on the protein level, only one study showed increased MMP-2 activity.^55^ Thus, the MMP/TIMP balance might be shifted towards inhibition of ECM breakdown even whilst the MMP expression is increased. Remarkably, it appears that collagenases [MMP-1, -8, and -13] have not yet been studied thoroughly, while these are the only MMPs that can actually digest cross-linked collagens that accumulate during intestinal fibrosis. Thus, future studies should focus on the presence and activity of a broader range of MMPs and TIMPs, especially those involved in the degradation of cross-linked collagen. Since TIMP-1 can inhibit a broad spectrum of MMPs, it is logical to assume that the collagenase activity *in vivo* is inhibited. Indeed, MMP-1 activity was shown to be similar in fibrotic CD mucosa compared to controls. Higher expression of MMPs [e.g. MMP-1, -2, and -12] in fibrotic lesions might also have a pro-fibrotic effect since they also facilitate cell migration and chemokine processing.^14^ For instance, MMP-1 and -2 can activate latent TGF-β and MMP-9 and -12 can inactivate plasminogen. Future studies should elucidate how and when these MMPs act as pro- or anti-fibrotic factors. Expression of MMPs and TIMPs appears different in muscularis compared to mucosal tissue, but this is not been thoroughly studied. While muscularis and submucosa are probably more involved in intestinal fibrosis, the role of MMPs is still more extensively studied in mucosal tissue. In summary, new studies should be initiated that will generate knowledge about the pro- or anti-fibrotic role of MMPs in intestinal fibrosis. Interesting targets are MMPs that can activate latent TGF-β1, thereby promoting fibrogenesis [e.g. MMP-2, -9, -13, and -14^110,111]^; MMPs that can process plasminogen, thereby preventing activated plasminogen [plasmin] for processing ECM and activating pro-MMPs [MMP-2, -7, -9, -12, and -19^33,100,112]^; MMPs that specifically process cross-linked collagens [MMP-1, -8, and -13]; and last, but not least, inhibitors of MMPs [TIMPs, α2-macroglubulin, thrombospondin ½, and RECK^17]^. Future *in vivo* studies should be aware of the importance of [1] patient characteristics [e.g. medication and underlying disease], [2] selection of proper controls, [3] specifying the sample/tissue source [layer and region], and [4] antibody selection and specification.

Improvements in research techniques and analysis methods, for example in antibody specificity and single-cell omics, should help us to unravel the complex and diverse functions of MMPs in intestinal fibrosis, which could potentially lead to biomarkers and novel drug targets.

**Key questions to be answered in new studies**

Which MMPs and TIMPs are differently expressed/active in fibrotic intestinal tissue?
How are MMPs and TIMPs differently expressed in the several intestinal layers and cells?
Which MMPs are considered pro- or anti-fibrotic in fibrotic intestine?
How can we study MMP and TIMP activity and function in a relevant experimental setting?
How can we steer MMP and TIMP activity towards fibrosis resolution without causing tissue damage?

## Data Availability

No new data were generated or analysed in support of this review.
